# Comparative durability of nevirapine versus efavirenz in first-line regimens during the first year of initiating antiretroviral therapy among Swaziland HIV-infected adults

**DOI:** 10.11604/pamj.2013.15.5.1889

**Published:** 2013-05-03

**Authors:** Simbarashe Takuva, Denise Evans, Khangelani Zuma, Velephi Okello, Goedele Louwagie

**Affiliations:** 1 Clinical HIV Research Unit, Department of Internal Medicine, School of Clinical Medicine, Faculty of Health Sciences, University of the Witwatersrand, South Africa; 2 School of Health Systems and Public Health, Faculty of Health Sciences, University of Pretoria, South Africa; 3 Health Economics and Epidemiology Research Office, Department of Internal Medicine, School of Clinical Medicine, Faculty of Health Sciences, University of the Witwatersrand, South Africa; 4 HIV/AIDS, STIs and TB, Human Sciences Research Council, Pretoria, South Africa; 5 Swaziland National AIDS Programme, Ministry of Health and Social Welfare, Mbabane, Swaziland

**Keywords:** Tolerability, Toxicity, Efavirenz, Nevirapine, Antiretroviral therapy, Resource limited setting, Swaziland

## Abstract

Nevirapine (NVP) and Efavirenz (EFV) have generally comparable clinical and virologic efficacy. However, data comparing NVP durability to EFV are imprecise. We analyzed cohort data to compare durability of NVP to EFV among patients initiating ART in Mbabane, Swaziland. The primary outcome was poor regimen durability defined as any modification of NVP or EFV to the ART regimen. Multivariate Cox proportional hazards models were employed to estimate the risk of poor regimen durability (all-cause) for the two regimens and also separately to estimate risk of drug-related toxicity. We analyzed records for 769 patients initiating ART in Mbabane, Swaziland from March 2006 to December 2007. 30 patients (3.9%) changed their NVP or EFV-based regimen during follow up. Cumulative incidence for poor regimen durability was 5.3% and 2.7% for NVP and EFV, respectively. Cumulative incidence for drug-related toxicity was 1.9% and 2.7% for NVP and EFV, respectively. Burden of TB was high and 14 (46.7%) modifications were due to patients substituting NVP due to beginning TB treatment. Though the estimates were imprecise, use of NVP - based regimens seemed to be associated with higher risk of modifications compared to use of EFV - based regimens (HR 2.03 95%CI 0.58 - 7.05) and NVP - based regimens had a small advantage over EFV - based regimens with regard to toxicity - related modifications (HR 0.87 95%CI 0.26 - 2.90). Due to the high burden of TB and a significant proportion of patients changing their ART regimen after starting TB treatment, use of EFV as the preferred NNRTI over NVP in high TB endemic settings may result in improved first-line regimen tolerance. Further studies comparing the cost-effectiveness of delivering these two NNRTIs in light of their different limitations are required.

## Introduction

Non-nucleoside reverse transcriptase inhibitor (NNRTI)-based antiretroviral therapy (ART) has been the most affordable regimen for HIV-infected patients in resource limited settings. Combination ART with either efavirenz (EFV) or nevirapine (NVP), both NNRTIs, and two nucleoside reverse transcriptase inhibitors (NRTI) is recommended by the World Health Organization (WHO) as first-line therapy in resource-limited countries [1]. NVP and EFV have generally comparable clinical and virologic efficacy [2–4]. The 2NN trial which compared EFV with NVP, both given with stavudine (d4T) and lamivudine (3TC) showed that virologic responses were similar for both drugs but compared with EFV, NVP was associated with greater toxicity and but similar in efficacy to EFV [4]. Limited data from routine clinical practice in resource limited settings, are available that directly compare NVP to EFV durability among HIV-infected patients [2, 5]. Clinical trial data comparing NVP durability to EFV are imprecise [4, 6, 7]. Additionally, most data are based on patient populations from developed countries who exhibit different clinical and demographic characteristics in comparison to patients initiating ART in resource limited settings. Data from these patient populations may not be generalizable to different clinical settings.

Where treatment options are limited, strategies to achieve maximum benefit from the available first-line regimens are vital and understanding which regimens are most tolerated by patients is therefore crucial. We set out to compare the durability of NVP to EFV in standard first-line regimens among HIV-infected treatment naïve patients initiating ART and followed up to a year at a large urban clinic in Mbabane, Swaziland.

## Methods

### Study setting and population

The Mbabane Government Hospital Antiretroviral Therapy Unit has previously been described [9]. The hospital is located in a peri-urban setting and houses the largest antiretroviral therapy outpatient clinic in Swaziland. In this retrospective analysis of cohort data, we included largely ART naïve (except prior exposure to short course antiretrovirals for Prevention of Mother to Child Transmission of HIV), HIV infected persons > 18 years of age and patients initiated on a standard first-line regimen according to WHO 2006 ART guidelines from 01 March 2006 to 31 December 2007 [1]. Patients were preferably initiated onto NVP- based regimens, with lamivudine (3TC) and either stavudine (d4T) or zidovudine (AZT). Patients on TB treatment or with clinical suspicion of a TB diagnosis at the time of ART initiation were started on EFV-based regimens.

### Data collection and Statistical analysis

The primary outcome was poor regimen durability. We defined poor regimen durability as a physician initiated modification from either a NVP-based or EFV-based ART regimen due to any reason (reasons could have broadly included either drug toxicity, drug contraindication, treatment failure or drug stock-outs). Drug toxicity based modifications were based on treatment guideline recommendations and were also considered after patient complaints [1].

Patient data was extracted from a computer-based system, patient files and pharmacy refill booklets. All patients were followed up until treatment modification, loss to follow-up, transferred out to another facility or administrative censoring after 12 months of follow-up. Since we did not have death data available for this cohort, a significant proportion of the patients classified as LTFU could have died.

We calculated cumulative incidence of the NNRTI drug modifications by end of the 12 month follow up period. Multivariate Cox proportional hazards models were further employed to estimate the risk of poor regimen durability for the two regimens. Separate multivariate analyses were also performed to estimate risk of poor regimen durability caused by drug-related toxicity alone. All analyses were performed using STATA v.11 (STATA Corp., College Station, TX, USA). Lastly, in order to understand how patients who were lost to follow up (LTFU) could have biased our data, we performed sensitivity analysis that assumed that all LTFU were failure events. Patients missing 3 consecutive clinic visits, a month apart and were unable to be contacted were defined as LTFU.

### Ethics

Permission to access and use data for this study was granted by the ethics committees of the Swaziland Ministry of Health and Social Welfare and the Faculty of Health Sciences, University of Pretoria. This study drew data from a previous study that had assessed reasons for treatment change among patients initiating ART [9].

## Results

### Characteristics of the study cohort

Between 01 March 2006 and 31 December 2007, 824 patients fulfilled the inclusion criteria. Among these, 55 patients were excluded for missing data leaving a total of 769 patients for analysis. The majority of patients were initiated on to a NVP-based regimen (n = 578, 75.2%) and patients on NVP were more likely to be initiated on a d4T/3TC NRTI backbone compared to those initiated on an EFV-based regimen (48.8% vs. 19.9%; p < 0.001). Patients initiating NVP-based regimens were more likely to be female (NVP-based: 417/578; 72.2% vs. EFV-based: 95/191; 49.7%; p < 0.001). CD4 cell count, WHO clinical stage, weight and age were largely similar among the two groups at the time of ART initiation (Table 1). At the end of the 12 month follow-up period, 17.2% (132/769) patients were LTFU and these patients were more likely to be on an EFV-based than a NVP-based regimen (22% vs. 15.6%, p = 0.04).

**Table 1:** Baseline, follow-up characteristics and NNRTI regimen modifications among the 769 patients initiating ART at the Mbabane Government Hospital ART Unit

Characteristics	NVP- based, n (%)	EFV- based, n (%)	p-value
Total	578 (75.2%)	191 (24.8%)	
**Gender**			
Female, n (%)	417 (72.2%)	95 (49.7%)	<0.001*
Male, n (%)	161 (27.9%)	96 (50.3%)	
**WHO stage**			
II/II, n (%)	259 (47.8%)	80 (44.9%)	
III/IV, n (%)	283 (52.2%)	98 (55.1%)	0.5*
CD4 count, median (IQR)	119 (67 - 187)	102 (56 - 171)	0.06¶
Weight, median (IQR)	62 (55 - 69.5)	60 (55 - 68)	0.67¶
Age, median (IQR)	35.3 (30.7 - 42.9)	36.9 (32.1 - 43.2)	0.19¶
**Regimen**			
d4T/3TC backbone, n (%)	282 (48.8%)	38 (19.9%)	<0.001*
AZT/3TC backbone, n (%)	296 (51.2%)	153 (80.1%)	
LTFU, n (%)	90 (15.6%)	42 (22%)	0.04*
Poor regimen tolerability to either NVP or EFV, n (%)	26 (4.5%)	4 (2.1%)	0.16*
**Reasons forpoor regimen durability**			
**Contraindication**			
TB treatment	14	0	
Drug shortage	2	0	
**Toxicity**			
Raised AST/ALT/hepatitis	4	0	
CNS disturbances	0	3	
Gynaecomastia	0	1	
Lactic acidosis	1	0	
Rash or hypersensitivity	4	0	
Treatment failure**	1	0	
Total modifications	26	4	

NNRTI, non-nucleoside reverse transcriptase inhibitor; NVP-Nevirapine; EFV-Efavirenz; d4T-stavudine; AZT-zidovudine; 3TC-lamuvidine; LTFU-Loss to follow-up; TB-tuberculosis; AST- Aspartate transferase; ALT- Alanine transferase; CNS- Central Nervous System.

* Pearson's Chi-square test used.

¶ Wilcoxon Rank-sum test used.

** viral loads were not done routinely but only for patients with clinical suspicion of treatment failure after at least 6 months on treatment.

### Main findings

A total of 30 patients (3.9%) had either a NVP- or EFV drug modification for any reason during this 12 month follow up period (NVP 4.5% (26/578), EFV 2.1% (4/191)). Two patients discontinued their regimen because of a stock out of NVP tablets as part of the fixed dose combination Triomune^TM^. One patient switched treatment (NVP/3TC/d4T) to a second-line regimen due to confirmed treatment failure. All 14 (14/26; 54%) discontinuations due to a drug contraindication were due to patients starting TB treatment whilst on a NVP- based regimen. A total of 13 (13/30; 43%) patients experienced poor regimen durability caused by drug-related toxicity alone - this excludes 14 due to TB treatment, 2 due to drug shortage and 1 due to treatment failure. Cumulative incidence for NVP or EFV drug modification regardless of reason was 5.3% (26/488) and 2.7% (4/149) for NVP- and EFV- based regimens, respectively. The cumulative incidence for NVP or EFV drug modification due to drug-related toxicity alone by end of the 12 months was 1.9% (9/471) and 2.7% (4/149) for NVP- and EFV-based regimens, respectively. The reasons for these modifications are shown in Table 1.

In Cox proportional hazards models adjusted for age, gender, baseline CD4 cell count and WHO stage, the estimates suggest that being on a NVP- based regimen may be associated with increased risk of poor regimen durability compared to a EFV - based regimen though the imprecise nature of the estimate does not allow us to infer the association with confidence (HR 2.03, 95%CI 0.58 - 7.05). In analysis examining poor regimen durability due to drug related toxicity modifications, a NVP- based regimen seemed to have a small non-significant advantage over a EFV-based regimen. These estimates were also imprecise (HR 0.87, 95%CI 0.26 - 2.90). Results are shown in Table 2.

**Table 2:** Tolerability of nevirapine versus efavirenz based regimen as measured by treatment modifications among 769 patients initiating ART at the Mbabane Government Hospital ART Unit

	All modifications			Toxicity based modifications		
Regimen	Cumulative incidence	Hazard Ratio	95% CI	Cumulative incidence	Hazard Ratio	95% CI
NVP based regimen (n = 578)	5.3%	2.03	0.58 – 7.05	1.9%	0.87	0.26 – 2.90
EFV based regimen (n = 191)	2.7%	1.0	reference	2.7%	1.0	reference

ART – antiretroviral therapy; NVP - Nevirapine; EFV - Efavirenz; 95% CI – 95% confidence interval. Hazard Ratios adjusted for age, baseline CD4 cell count, WHO stage and gender

### Sensitivity analysis

In order to understand how the missing outcomes in the LTFU data could have affected our outcome estimates, we additionally conducted a sensitivity analysis in which we assumed that all LTFU patients actually had a NNRTI drug modification. In this analysis, there was no significant difference in outcome between the two regimens (HR 0.93, 95%CI 0.65 -1.44). This result may suggest that if LTFU did affect our main estimates, this bias was minimal.

## Discussion

In this observational study in which patients were followed up to 12 months after initiating first-line ART in Mbabane, Swaziland, the hazard estimates suggest that being on a NVP- based regimen may be associated with increased risk of poor regimen durability compared to a EFV - based regimen though the imprecise nature of the estimate does not allow us to infer the association with confidence. In further analysis examining poor regimen durability due to drug related toxicity modifications, a NVP- based regimen seemed to have a non-significant small advantage over an EFV-based regimen though these estimates were also imprecise.

It is important to note that all regimen changes due to drug contraindication were due to patients discontinuing NVP due to a tuberculosis (TB) diagnosis. This resulted in a far much higher proportion of discontinuations of NVP compared to EFV (twice as much for all types of modifications). According to local guidelines, concomitant use of NVP with rifampicin containing TB treatment is contraindicated as there is a potential of drug-drug interactions and increased toxicity [1]. In accordance with local HIV treatment guidelines, patients initiating ART were only put on NVP if they had no clinical suspicion of TB diagnosis or had begun TB treatment or had no other contraindication to NVP. However, the significant rate of NVP modifications due to TB diagnosis after ART initiation clearly emphasizes the high burden of both undiagnosed TB and incident TB in this setting among patients initiating ART [10]. This raises the need for more aggressive TB screening in patients initiating ART therefore aiding earlier identification of this endemic mycobacterium disease. Due to the high burden of TB (including undiagnosed TB) and frequent late diagnosis, it seems plausible to recommend EFV as the preferred NNRTI in order to reduce the rate of treatment interruptions seen when NVP is used as a first-line regimen choice. In terms of toxicity related durability, the toxicity profile of both NNRTIs was comparable with NVP having a small non-significant advantage over EFV. This finding is in contrast mostly to studies conducted in high-income and middle-income settings which show that NVP has a much poorer toxicity profile compared to EFV [3, 4]. Compared to patients from resource limited settings, high-income and middle-income patient populations mostly initiate ART at higher CD4 cell counts and higher CD4 counts at initiation have been associated with increased risk of NVP toxicity [8]. This has resulted in the cautious use of NVP in these clinical settings.

Our study has a major strength of the use of routine clinical data collected from a real life clinical setting. However, the findings of this study should be considered alongside its limitations. We did not collect comprehensive information of concurrent non-HIV clinical conditions that may increase the risk of drug modification i.e. diabetes, epilepsy, resulting in unmeasured confounding of our findings. Additionally, because of the retrospective nature of this data, precautions could not have been taken to ensure that any chosen drug combinations at treatment initiation or modifications after initiation could have resulted from sub-optimal prescribing practices rather than genuine intolerance of the drug by patients. Furthermore, the small number of outcome events and small sample size may have compromised the power of the study in order to detect sizeable differences. Finally, the effect of loss to follow up which may have included deaths were not considered in the main analysis. While this may have introduced selection bias into the estimates presented, sensitivity analyses suggested minimal bias.

## Conclusion

In conclusion, we observed an increased risk of modifying NVP in initial first line regimens compared to that of EFV in the first 12 months of treatment initiation. A significant proportion of patients modifying ART did this due to starting TB treatment. Use of EFV as the preferred NNRTI over NVP in settings were TB is endemic may result in improved first-line regimen durability. Better screening methods for TB should also be made readily available in such settings so that better tolerated regimens are initiated. Additional studies should focus on comparing the cost-effectiveness of delivering these two NNRTIs in light of their different limitations. Improved initial ART regimen durability should be an additional vital treatment aim as this will increase the likelihood of achieving sustained viral response and, at the same time, preserving future treatment options.
